# Digital outpatient health solutions as a vehicle to improve healthcare sustainability—a United Kingdom focused policy and practice perspective

**DOI:** 10.3389/fdgth.2023.1242896

**Published:** 2023-09-26

**Authors:** Matthew R. D. Brown, Matthew Knight, Christopher J. Peters, Simin Maleki, Ali Motavalli, Bahman Nedjat-Shokouhi

**Affiliations:** ^1^ Medefer, London, United Kingdom; ^2^ The Royal Marsden Hospital NHS Trust, London, United Kingdom; ^3^ West Hertfordshire Teaching Hospitals NHS Trust, Watford, Hertfordshire, United Kingdom; ^4^ Department of Surgery and Cancer, Imperial College London, London, United Kingdom

**Keywords:** digital healthcare, sustainability, environment, greenhouse gases, economy

## Abstract

**Introduction:**

In the midst of a global climate emergency and with health care systems across the world facing extreme pressure, interest in digital approaches as a potential part-solution to these challenges has increased rapidly. The evidence base to support the role that digitalization can play in moving towards more sustainable models of healthcare is growing, as is the awareness of this key area of healthcare reform amongst policy makers, clinicians and the public.

**Method and Results:**

In this policy and practice review we explore four domains of healthcare sustainability-environmental, economic, and patient and clinician, delineating the potential impact that digitally enabled healthcare can have on each area. Real-world examples are provided to illustrate the impact individual digital interventions can have on each pillar of sustainability and demonstrate the scale of the potential benefits which can be achieved.

**Discussion:**

Digitally enabled healthcare solutions present an approach which offer numerous benefits, including environmental sustainability, economic benefits, and improved patient experience. There are also potential drawbacks such as the risk of digital exclusion and the need for integration with existing technology platforms. Overall, it is essential to strike a balance between the benefits and potential drawbacks of digital healthcare solutions to ensure that they are equitable, effective, and sustainable.

## Introduction

As health systems across the globe face unprecedented pressure due to the COVID-19 pandemic and subsequent recovery phase, novels ways of delivering healthcare must be identified and deployed. Furthermore, there is an ever-increasing drive towards delivering healthcare in a sustainable manner. Whilst sustainability may have once predominantly been associated with minimising environmental impacts of an activity, the term has now broadened to encompass the well-being of patients, staff and an organisation's long-term viability (1).

As a disruptor, the COVID-19 pandemic accelerated the adoption and recognition of digital health solutions as a viable means to deliver healthcare services both amongst providers and patients (2). When healthcare is compared to other industries, the adoption of digital solutions has historically been low. This situation has arisen due to numerous barriers including data security concerns, the cost and complexity of project development and implementation, a historic lack of demand from patients and a litany of failed IT programmes including the NHS National Programme for IT (NPfIT) estimated to have cost in excess of £10 billion (3).

Digital health solutions embody a range of different interventions. Whilst the definition remains relatively ambiguous, it is accepted that it includes wearable devices and sensors, telemedicine, electronic patient record systems and the application of data-science informatics and artificial intelligence to healthcare (4). In combination, this technology and associated changes in practice and patient engagement required represent a significant transformation in the way in which healthcare is and can be delivered. Whilst short-term change and remodelling of services may be disruptive, the healthcare ecosystem which can be created with functioning digital health solutions at their core is predicted to offer significant benefits negating transition-related disruption.

Such is the perceived potential that in 2020 the World Health Organisation (WHO) launched a digital health strategy aiming to advance the implementation of national digital health strategies, strengthen governance, promote collaboration and knowledge transfer and advocate people-centred healthcare facilitated by digital health (5).

In parallel to this, the United Kingdom's (UK) National Health Service (NHS), in the latest iteration of its Long Term Plan (6), makes a clear commitment to what it terms digitally-enabled care being developed and adopted as an integral feature of standard of care for the majority of patients. This approach to the digitalisation of health provision within the UK is further supported by the recommendations of the Royal College of Physician's 2018 report *Outpatients: the future—adding value through sustainability* which states that “all outpatient care pathways should aim to minimise disruption to patients” and carers’ lives’ and that “there is good evidence that new technologies will support innovation in outpatient services” (7). The NHS Long Term Plan suggests redesigning outpatient services and removing up to the third of face-to-face outpatient visits by 2024 saving the NHS £1.1 billion.

Part of the challenge faced by organisations aiming to adopt new technology to assist in the provision of clinical services is providing clinicians with robust evidence of benefit. This paper aims to delineate a number of sustainability-related domains for which there is evidence that digital health solutions can contribute. Furthermore, it will provide illustrations in the form of case studies from a novel virtual hospital service, Medefer. Medefer is a digitally enabled healthcare solution which, through its software platform enables referrals from primary care to be reviewed and managed online by a team of UK-registered specialty consultants. This approach results in patients often not having to wait for an in-person hospital appointment resulting in many conditions being managed completely remotely. Medefer is one of the largest providers of digital secondary care services in the UK across a broad range of specialties and, as such, provides an unrivalled case study in the way in which digital outpatient services can impact on aspects of sustainability within the UK.

In general, sustainability refers to three distinct areas: social, economic, and environmental—known as the three pillars of sustainability (8). When considered in the context of healthcare, sustainability gains can be viewed as encompassing 4 broad domains which are directly related to the three pillars, namely, environmental, economic, patient and clinician related. Whilst the concept is relatively expansive in what it includes, in all the domains sustainability gains should satisfy the definition of “after a defined period of time, a program, clinical intervention, and/or implementation strategies continue to be delivered and/or individual behaviour change is maintained; the program and individual behaviour change may evolve or adapt while continuing to produce benefits for individuals/systems” (9). It is interesting to note that the concept of healthcare sustainability is to a degree independent of the clinical setting in which it occurs and is scalable, representing significant potential system-wide improvements. Sustainability, the individual domains and the influence digitally enabled healthcare has on them are outlined and explored in further detail below.

### Environmental sustainability

#### Airborne particulates and greenhouse gases

It is estimated that emissions linked to the NHS currently contribute 5% of the UK's annual carbon footprint, and in October 2020, the NHS committed to reaching net zero carbon emissions by 2045 (10). A proportion of these greenhouse gas (GHG) emissions are transport-associated and generated by patients attending healthcare facilities for face-to-face consultations, investigations, and imaging.

A clear benefit of using digital solutions is the ability of healthcare provision to occur remotely, thereby eliminating or significantly reducing the requirement for a patient, and often a family member or carer, to travel to healthcare facilities. It has been estimated that annually, patient's travel contributes 5% of the NHS's carbon footprint as well as generating approximately 118 t of particulate matter (PM) air pollutants and 2,602 t of nitrogen oxides (NOx) (11). PM comprise smoke, fumes, soot, and other combustion residue and natural substances such as dust. Inhalable particles range in size between aerodynamic diameter 1 μm (PM1) and 10 μm (PM10) which can penetrate the alveolar gas-exchange regions of the lungs, and may be specifically related to health effects leading to premature death (12).

An illustration of the impact that digitally enabled healthcare can make on reducing harmful vehicle emissions is provided by Medefer's operations. In the financial year 2021–2022 a total of 91,926 individual patient reviews were conducted digitally [data extracted from Microsoft Power BI (Microsoft Inc, Redmond, WA, USA)]. It is estimated by the NHS's sustainable development unit that the average trip to attend hospital involves a round trip of 34 km (11). Using this figure, it can be calculated that by conducting these reviews remotely, an estimated total of 3,125,484 kilometres of car travel was rendered unnecessary. The equivalent of driving around the world 245 times.

This in turn resulted in a reduction in PM10 production of 21.87 kg and PM2.5 production of 9.4 kg based on UK National Atmospheric Emissions Inventory (NAEI) figures of average car production all road types for PM10 brake wear EF of 7.0 mg km^−1^ veh^−1^ and for PM2.5, the corresponding value was 3.0 mg km^−1^ veh^−1^ (13). Whilst this reduction in particulate release is clearly beneficial to the environment and population in general, it is particularly relevant in areas in close proximity to healthcare facilities where individuals with pre-existing health conditions who make them more susceptible to particulate induced harm (14).

An activity's carbon footprint represents the sum of greenhouse gas emissions associated with that process and is expressed in carbon dioxide equivalents (CO2e). For outpatient appointments this can be calculated using the following.

Carbonfootprint(kgCO2e)=activityorresource×GHGemissionsfactor

If the NHS Sustainable Development Unit's figure for average GHG emissions of 5.8 kgCO_2_e per hospital trip is used, the reduction due to the elimination of the requirement to travel to and from appointments equates to a saving of 533,170 kgCO_2_e, or 540 transatlantic flights per annum—representing a significant reduction in GHG emissions achieved by a single digital health intervention. If such an approach was deployed across the UK's NHS then the potential savings reductions in GHG would clearly be even more sizeable.

#### Impact on facilities and estate

In addition to the clearly defined environmental benefits of conducting remote consultations through a “virtual hospital”, is that the footfall through healthcare facilities is reduced. This is particularly prescient when social distancing measures are employed as a risk reduction measure for airborne pathogen spread. Fewer patients physically within a healthcare facility reduces both interaction between patients and clinic staff (thereby reducing the risk to frontline staff) but also reduces the contact patients may have with each other, for example when waiting in seating areas. In addition to the reduction in footfall in clinical facilities and the clear impact this has on capacity, there is increased awareness of energy expenditure associated with running “bricks and mortar” healthcare establishments of which outpatient facilities comprise a substantial proportion. This is of particular relevance in the context of significantly inflated energy costs. In the year 2020–21 the total energy usage from all energy sources across the NHS estate was 11.4 billion kWh (15), approximately equivalent to the domestic energy usage of 1 million UK homes, therefore any reduction in the requirement for physical healthcare facilities will help ameliorate this demand benefiting the environment but also reduce costs.

### Economic sustainability

#### Reduction in GVA loss

The process of an individual patient, often accompanied by a carer, attending an outpatient consultation has an impact on local economic productivity due to tangible reductions in the time employees and carers are present at work on the day of their appointment. This impact is amplified by the sheer numbers of patients who attend an outpatients’ appointment each year—there were 78 million attendances in 2020–2021 (16).

Recent work performed by the Midlands and Lancashire Commissioning Support Unit has sought to develop a methodology to quantify the impact of outpatient appointment attendance on Gross Value Added (GVA)—a measure of economic impact defined as the additional income to an area generated from economic activity and the production of goods and services (17). Using a combination of estimated time for journey to and from appointments (56 min), average time spent waiting for appointments (51 min) actual appointment time (20 min) and an annual output per hour worked across the UK's whole economy in 2021 equal to £40.02 (18), average GVA loss per appointment was calculated to equal £84.44.

Not all patients attending clinic appointments will be employed either due to them being too young to work or being retired, additionally not all the working age population are employed. To adjust the figures accordingly the total number of appointments attended by patients between the age of 20 and 64 can be ascertained from NHS digital's hospital outpatient activity data 2020–21 (40,873,275) and then multiplied by the UK employment rate in 2021 (96.4%) to give a total of 39,401,837 appointments. Therefore, by using the adjusted attendance figures and estimated loss of GVA, it can be calculated that approximately £3.4 billion was lost from the UK economy as a direct consequence of outpatient appointments attended in 2021–2022.

Clearly when remote consultations are conducted, the requirement for the patient to travel to hospital is eliminated, this reduces the need for patients to take time off work and in general renders the clinical review more efficient. If the total time taken for a remote consultation is assumed to be on average the same as a face-to-face consultation (20 min) but the travel and wait times are eliminated, the GVA loss per appointment would be £28.15, a significant reduction from the £84.44 loss seen with a traditional model of care. The beneficial impact on the economy of these efficiency savings can be illustrated by a year's worth of Medefer review figures. In 2020–21 91,926 patient reviews were conducted. If these were all face-to-face appointments and the same proportion (50%) of patients were assumed to be in employment, then this process would cost the economy £3.88 million pounds in lost productivity. Conversely, the 91,926 patients managed using the Medefer assessment pathway only required 10,369 virtual consultations, the rest of the clinical decision-making being undertaken with no direct patient contact, relying instead on previously provided clinical information. This process, again assuming similar employment rates, would result in only £140,000 being lost from the economy, representing a productivity gain of over £3.5 million pounds.

#### Reduction in outpatient appointment non-attendance (DNA)

Non-attendance at outpatient appointments has serious economic consequences. In the financial year 2021–22, 6.5% or 7.8 million out of 122.3 million outpatient appointment episodes booked ended in a “Did Not Attend” (DNA), comprising 1 in every 12 appointments. This represents an increase of 15.6% from the previous year (19).

Outpatient services account for approximately 7% of the NHS budget. Considering each outpatient appointment is estimated to cost the NHS approximately £120 (20), the total cost would be close to £936 million. Causes of non-attendance vary substantially between organisations and regions and are a combination of patient characteristics and healthcare setting factors. Common patient-related causes for patients missing their appointments include forgetting about it or patients feeling they no longer need a review. Additional contributory factors include reasons outside of patient's control such as not being aware of the appointment, receiving incorrect information regarding the appointment, having difficulty cancelling or rescheduling the appointment, not receiving a reminder after being on a waiting list for a long time, difficulty arranging a carer, transport, and financial issues.

Providers need to identify the predominant local causes and understand issues and barriers faced by particular demographic groups. The aim would be to reduce the number of DNAs leading to clearing backlog and reduced waiting time. A focus on better communication, reduction of unnecessary follow ups, more personalised care and the prediction of demand enables resources to be invested in faster more modern diagnostics and other much needed healthcare capacity.

Over 2021–2022, the highest proportion of non-attenders were aged between 30 and 39, highlighting that current outpatient services may not be most suitable for the working population or those with childcare commitments. Technological solutions can be effective in addressing some DNAs, a pilot of Skype-based virtual consultations in Newham's DAWN scheme reported a reduction in DNAs from 30%–50% to 16.8%, demonstrating that the shift to virtual consultations increases the convenience and accessibility of appointments (17). Appointment reminders have also been shown to be effective in reducing the number of DNAs, this approach can be combined with digital solutions such as patient portals to reduce DNAs by providing a route for accessing and sharing patient information electronically. This in turn releases clinical and administrative time, enables patient education and encourages patients to be active participants in their care.

### Clinician sustainability

#### Professional sustainability and resilience

Maintaining the morale and motivation of clinical teams working in the recovery phase of the COVID-19 pandemic is a major priority for healthcare providers. This is important if the significant backlog of clinical work that exists is to be tackled. The incidence of clinician “burnout” and disengagement is increasing (21) for a number of reasons which include poor working conditions and job satisfaction, onerous administrative burdens and high clinical work load. Whilst the introduction of digitally-enabled healthcare processes may intuitively be thought to mitigate the risk of burnout, evidence around this area is mixed with some data suggesting that electronic health record (EHR) systems, when poorly deployed, may increase burnout rates (22). Conversely, recent work has shown that those clinicians who use digital health solutions were significantly more likely to report higher job satisfaction and better work-life balance than those clinicians who did not (23). With the rapid adoption of digitally enabled healthcare approaches in response to the COVID-19 pandemic, it is clear that in order to ensure development of high quality solutions and excellent outcomes, clinician engagement, training and support must be recognised and provided (24), minimising the risk of poorly deployed systems which may further contribute to clinician burnout.

In order for a healthcare provider to remain viable, a critical mass of clinical expertise and services is required. This situation is becoming challenging to maintain with a rising number of unfilled clinician posts within NHS hospitals—a situation predicted to worsen as consultants in their 50s retire and reduced numbers of new consultants are trained due to a retention crisis amongst junior doctors (25). One solution to this situation is to develop systems whereby it is possible provide the necessary specialty-specific expertise remotely, enabling a broader range of clinical expertise to be offered to local populations.

### Patient experience

#### Patient satisfaction

As outlined above, the process of attending an outpatient consultation is often a time-consuming and unpleasant experience for the patient involved. This, combined with the organisational and logistical challenges involved in arranging appointments, undoubtedly contributes to the large number of non-attended outpatient appointments which occur each year [nearly 5.5 million in 2020–21 (16)].

Digital solutions offer significant and compounded benefits which are distributed along the clinical pathway and impact on the system, patient, and clinical stakeholders. From a patient perspective, digitalisation of outpatient processes results in increased efficiency; the consultation can be conducted at a timepoint more favourable to the individual patient, travel time is eliminated and the risk of a lengthy wait to be reviewed is reduced. The use of the consultation time window is also optimised as investigations and previous correspondence pertinent to the case can be collated and checked in advance. Additionally, relevant resources and information can be signposted during the consultation. Access to clinical pathway dashboards and portals allows patients to track their progress, enabling heightened engagement with their management plan and a greater understanding of how their condition is being investigated and treated, overall, the combination of these factors results in increased patient satisfaction (26) and evidence that such engagement can change patient's self-efficacy in a positive fashion (27).

During the COVID-19 pandemic, the importance of digital health services increased, partly in response to the requirement to manage the disruption caused by increasing number of unwell patients. In this regard, digital healthcare interventions helped healthcare services to tackle the pressure placed on their capacity, but also enabled patients to participate in their treatment plan virtually. In order to assess the perception of benefit amongst patients, a systematic review study was conducted evaluate patient satisfaction with digital health services during pandemic worldwide (28). Patients of various nations in 34 papers from total 42 publications were satisfied with the digital health provided services. This highlights the importance of the shift to the digitalized healthcare era. High patient satisfaction with digital health solutions may, in part, be due to the reshaping of the patient-physician relationship that they permit. By giving more authority to the patient in the process of decision making, patients have become a more decisive part of formulating a treatment plan rather than depending solely on doctor's decisions (29).

#### Patient self-efficacy

Evidence shows that digital interventions can change patient's self-efficacy in a positive fashion (27). For example, Olander demonstrated that four main digital-based techniques including action planning, prompt self-monitoring of behavioural outcome, plan social support/social change and time management had a positive impact on self-efficacy (30). In another study which was conducted to evaluate the effect of the Internet and mobile-based interventions (IMIs) on youths with chronic mental health issues, a positive effect on self-efficacy was found in favour of IMIs (31). In other words, by improving self-efficacy, digital health solutions can provide effective and safe interventions in three main domains: promoting healthy behaviours such as smoking cessation (32), providing remote access to specific types of treatment such as computerized cognitive behavioural therapy, and improving outcomes in patients with chronic physical and mental health conditions (33).

It is important to note that while digital interventions provide substantial opportunities to interact with intervention receivers in different ways, this mode of delivery may limit the human-based contact between the intervention deliverer and recipient (27). Moreover, due to the rapid pace of innovations in the field of healthcare, it is necessary to ensure that digital interventions are kept relevant and up to date. However, rapid changes in digital health offerings may prove difficult for patients to adapt to in short periods of time (34). Furthermore, effective engagement with web-based interventions and monitoring services requires specific training for patients which might not be feasible on a larger scale. For instance, there was no significant improvement in specified parameters such as blood pressure or HbA1C levels if participants had difficulties with registration process or inserting their health information (35).

#### The “clinical concierge” and long term conditions

As the complexity of modern healthcare and the rates of multimorbidity increase, and a more personalised approach to healthcare is adopted across numerous medical specialties, an individual patient's healthcare management is becoming ever more expansive and complicated. This situation increases the risk of inadvertent harm occurring from factors including polypharmacy (36), lost correspondence, non-attendance to clinical reviews and missed diagnostic or surveillance investigations (37). One feature of digitally enabled healthcare is the ability to provide support to patients to attempt to minimise the complexity of pathways, enhance engagement with the clinical plan and prompt patients to attend investigations and appointments. This approach is supported by published data showing improved outcomes across a range of conditions including COPD (38), diabetes (39) and the perioperative journey (40). The applicability of these interventions is striking when deployed to help manage long-term conditions. Long-term conditions are defined as those conditions which cannot, at present, be cured but are controlled by medication and/or other treatment. With an ageing population the incidence and complexity of those patients living with long-term conditions (LTCs) is increasing.

Patients with long-term conditions are intensive users of healthcare services accounting for 50% of all GP appointments, 64% of outpatient appointments and 70% of all inpatient bed days. In total around 70% of the total health and care spend in England is attributed to caring for people with LTCs meaning that 30% of the population account for 70% of the spend (41). Digital healthcare has the potential to play a significant role in reducing healthcare resource utilization by patients with LTCs in the UK. Several studies and reports have highlighted the benefits of digital healthcare interventions in managing LTCs, which may help to reduce the burden on healthcare resources. A systematic review of telehealth interventions for patients with LTCs found that telehealth, which includes remote monitoring, teleconsultation, and tele-education, showed promising results in improving clinical outcomes, reducing hospitalizations, and enhancing patient self-management capabilities (42).

Digital technologies, such as wearable devices, mobile apps, and telemedicine platforms, can enable remote monitoring of vital signs, provide timely access to healthcare professionals, deliver personalized education, and support self-management, which can reduce the need for frequent hospital visits or emergency care for patients with LTCs. A randomized controlled trial conducted in the UK evaluated the impact of telehealth on healthcare utilization among patients with LTCs and found that telehealth reduced emergency hospital admissions by 45% and emergency department visits by 20% (43). The study also reported high patient satisfaction and improved quality of life among participants who received telehealth interventions.

## Discussion

As we face the realities of healthcare in the post-COVID world, it is clear that significant and systematic challenges exist to delivering services in an effective and sustainable fashion. Traditional models of healthcare provision now appear inadequate to meet the challenges of rising demand, reduced resources and environmental accountability and it is imperative that novel approaches are developed, deployed, and assessed. Digitally enabled healthcare solutions offer an attractive approach, which, if well integrated into existing clinical pathways and processes can provide numerous benefits distributed across the healthcare system and the local economy delivering a positive influence on the various pillars of sustainability. As outlined above these can be classified across several domains, from an environmental perspective, the main impact of digitalisation pivots on significant reductions in GHG and harmful particle emissions which can be easily quantified use the methods detailed, these emission reductions are important as they are focused in localities close to hospitals where patients who are susceptible to the detrimental effect of these substances are concentrated.

From an economic perspective the benefits of digitally enabled care are system wide with potential savings both for hospitals who experience reduced pressure and demand on their bricks and mortar facilities and reduce missed appointments and investigations alongside the beneficial effects on local economic productivity by minimising worker absenteeism. Finally digital approaches have been shown to improve patient experience and may be used to contribute to more sustainable models of care in health services which are operating under unprecedented levels of demand and pressure. There is additionally a major degree of overlap observed between these domains, amplifying the benefits. Due to the nascent nature of digital healthcare, the published evidence base supporting a number of these benefits is currently relatively limited. However, as this area of healthcare develops and matures, the quantity of relevant published research will inevitably expand aided by the fact that by its very nature digital healthcare provides ample opportunity to collect and analyse data.

It is also clear that areas of interest in this field include not just the positive impact of digitalisation but also the risks and potential shortfalls of such approaches and the optimal manner in which such digitally enabled solutions can be aligned with existing healthcare services. As the field of digital healthcare matures, learning and experience will help guide the effective adoption and deployment of digital health solutions as well as shape its future development.

Whilst the benefits of digital healthcare can be delineated relatively easily, there are also potential drawbacks which must be considered. Firstly, in order to engage with digital healthcare access to devices which support the relevant platforms is vital. Inequality in this area is entrenched and unsurprisingly aligned with the distribution of broader health inequalities in a population, namely socio-economic, geographic, and ethnic factors which are often combined. Other factors which may affect the uptake of digital therapeutics include the interoperability of the digital therapeutic with the broader health system, the robustness of the regulatory environment the product is being used in and the patient perception and expectation of the product (44). Restricted access to digitally enabled healthcare, a situation termed digital exclusion, results in a situation where those groups who are most susceptible to poor health are the least likely to engage with and benefit from digital health innovations. Approaches to counter this situation should be adopted as a public health measure with the aim to increase digital literacy amongst marginalised groups in society as well as broadening the access to relevant hardware—for example through the promotion of schemes such as community hubs which provide a number of services including support for individuals to get online.

Secondly, within the UK, the adoption of external digital solutions often requires integration with existing technology platforms operated by hospitals and other healthcare facilities. This situation poses a number of challenges including the legacy nature of some of the platforms, minimal financial resources to support deployments and the lack of trained information technology staff within the NHS to support such integration. Whilst the UK is not unique in presenting a challenging environment to the adoption of digital health solutions, other similar countries are more advanced in developing formalized mechanisms for the assessment and reimbursement of some digital health products (45), moves which align with the aims outlined by the WHO in its Global strategy on digital health 2020–2025 (5).

An interesting consideration is the role that digital health innovations could and do play in improving access to high quality healthcare in resource-poor areas of the world. It is estimated that there will be over 7 billion smart device subscriptions globally by 2028 (46). This situation provides a novel mechanism by which both health advice and direct healthcare can be provided to individuals and communities who may have historically struggled to access these resources. For example, unmet need in healthcare is particularly acute in rural areas of low- and middle-income countries which are often “under doctored” compared to urban areas.

Specific areas in which digital health solutions can be deployed in these settings include process optimisation such as digitising birth registration or improving the efficiency of healthcare systems, clinical pathway optimisation such as the implementation of triage tools and population-level applications such as disease monitoring and prediction (47).

However, as with all aspects of digital healthcare, barriers exist and there is a risk that inequalities may be further entrenched rather than being ameliorated. As with the distribution of doctors varying between urban and rural areas in low- and middle-income countries so the ability to access the internet is skewed towards urban centres and the wealthier subsets of the population. Target groups such as adolescents may be under-represented in terms of reliable internet access (48) whilst regulatory environments may not be sufficiently mature to ensure high-quality products are released and used.

The post-COVID clinical landscape presents significant challenges to healthcare systems around the world, including rising demand, reduced resources, and environmental accountability. Traditional models of healthcare provision are no longer adequate to meet these challenges, and novel approaches are needed. Digitally enabled healthcare solutions offer an attractive approach that can provide numerous benefits, including environmental sustainability, economic benefits, and improved patient experience. However, there are also potential drawbacks, including the issue of digital exclusion and the need for integration with existing technology platforms. As the field of digital healthcare continues to develop, there is a need for an expanded evidence base to guide effective adoption and deployment of digital health solutions, as well as to shape its future safe and effective development. Overall, it is essential to strike a balance between the benefits and potential drawbacks of digital healthcare solutions to ensure that they are equitable, effective, and sustainable.
